# A smart millimeter-wave base station for 6G application based on programmable metasurface

**DOI:** 10.1093/nsr/nwaf017

**Published:** 2025-01-16

**Authors:** Jun Wei Zhang, Zhen Jie Qi, Li Jie Wu, Qun Yan Zhou, Jun Yan Dai, Wan Wan Cao, Xiao Ge, Cheng Long Gao, Xinxin Gao, Si Ran Wang, Zheng Xing Wang, Li Fang Yao, Jun Wei Wu, Jia Nan Zhang, Tie Jun Cui, Qiang Cheng

**Affiliations:** Institute of Electromagnetic Space, and State Key Laboratory of Millimeter Waves, Southeast University, Nanjing 210096, China; Institute of Electromagnetic Space, and State Key Laboratory of Millimeter Waves, Southeast University, Nanjing 210096, China; Institute of Electromagnetic Space, and State Key Laboratory of Millimeter Waves, Southeast University, Nanjing 210096, China; Institute of Electromagnetic Space, and State Key Laboratory of Millimeter Waves, Southeast University, Nanjing 210096, China; Institute of Electromagnetic Space, and State Key Laboratory of Millimeter Waves, Southeast University, Nanjing 210096, China; Institute of Electromagnetic Space, and State Key Laboratory of Millimeter Waves, Southeast University, Nanjing 210096, China; Institute of Electromagnetic Space, and State Key Laboratory of Millimeter Waves, Southeast University, Nanjing 210096, China; Institute of Electromagnetic Space, and State Key Laboratory of Millimeter Waves, Southeast University, Nanjing 210096, China; Institute of Electromagnetic Space, and State Key Laboratory of Millimeter Waves, Southeast University, Nanjing 210096, China; Institute of Electromagnetic Space, and State Key Laboratory of Millimeter Waves, Southeast University, Nanjing 210096, China; Institute of Electromagnetic Space, and State Key Laboratory of Millimeter Waves, Southeast University, Nanjing 210096, China; Institute of Electromagnetic Space, and State Key Laboratory of Millimeter Waves, Southeast University, Nanjing 210096, China; Institute of Electromagnetic Space, and State Key Laboratory of Millimeter Waves, Southeast University, Nanjing 210096, China; Institute of Electromagnetic Space, and State Key Laboratory of Millimeter Waves, Southeast University, Nanjing 210096, China; Institute of Electromagnetic Space, and State Key Laboratory of Millimeter Waves, Southeast University, Nanjing 210096, China; Institute of Electromagnetic Space, and State Key Laboratory of Millimeter Waves, Southeast University, Nanjing 210096, China

**Keywords:** beamforming, millimeter-wave, programmable metasurfaces, smart 6G base station

## Abstract

The evolution of programmable metasurfaces has yielded many exciting electromagnetic (EM) phenomena and applications in both communities of physical and information sciences. Programmable metasurfaces, also known as reconfigurable intelligent surfaces or intelligent reflecting surfaces in wireless communications, have played important roles in enhancing signal coverage and transmission quality, and in building an artificially controlled communication environment. However, most of the realistic implementations are designed in the sub-6G band with a small array scale and 1-bit phase control ability, making the performance improvement not marvelous compared with the traditional solutions. Here, we propose a large-scale 2-bit millimeter-wave programmable metasurface to build an integrated smart base station framework for 6G communications. The meta-array is composed of 30 × 30 meta-elements, each with two embedded positive-intrinsic-negative (PIN) diodes. A dish-cone antenna is integrated with the metasurface to serve as the feeding source. A control board is designed to autonomously switch the working states of all of the 1800 PIN diodes based on a field-programmable gate array, enabling the individual adjustment of the EM responses of all meta-elements in the array. Through the deliberate arrangement of phase distribution on the surface, the array can undergo reconfiguration to achieve the desired EM functionalities. We take the programmable metasurface as the core to assist a millimeter-wave base station and validate its good performance for wireless communications in a realistic indoor scenario. Subsequently, we build a four-stream wireless communication scenario using four 30 × 30 arrays and demonstrate smart multi-user information transmissions with different positions. This work provides great potential for programmable metasurfaces to aid the development of novel and intelligent millimeter-wave base stations, offering valuable insights for advancing next-generation mobile communications.

## INTRODUCTION

Metasurfaces are 2D versions of metamaterials that are composed of artificial subwavelength structures that have demonstrated remarkable physical phenomena over the past two decades [1–8]. They have captured the attention of numerous scientists due to their outstanding capability in manipulating all fundamental parameters of incident electromagnetic (EM) waves, including amplitude, phase, frequency, wavevector and polarization [9–12]. Additionally, their natural properties of easy fabrication and assembly, ultra-thin thickness, low loss and low power consumption also contribute to their appeal in engineering applications. The evolution of metasurfaces has progressed through passive metasurfaces, digital coding metasurfaces, and programmable metasurfaces [12–15]. Passive metasurfaces primarily function for the static manipulations of EM waves, while digital coding and programmable metasurfaces mainly focus on the dynamic reconfigurability of the EM waves and environment, making them better suited for practical scenarios. The digital coding metasurfaces can achieve real-time dynamic modulation of EM waves when integrated with tunable devices, such as positive-intrinsic-negative (PIN) diodes, varactors, graphene and micro-electro-mechanical systems devices [12,13,16,17]. For example, digital codes ‘0’ and ‘1’ are respectively employed to represent the two opposite phases realized by a 1-bit metasurface. By collaborating with digital signal processors such as micro control units and field-programmable gate arrays (FPGAs), the state of tunable devices that are embedded in the meta-elements can be controlled by using the predesigned coding sequences to enable real-time functional reconfiguration. With the assistance of intelligent sensors, the metasurfaces further possess the ability to intelligently reconfigure brilliant and smart features. Numerous devices and phenomena have been developed based on the properties of metasurfaces, including EM cloaking, beam refraction, holographic imaging and filtering [18–23], especially in wireless communications [24–35].

In particular, the programmable metasurfaces have recently aroused many discussions in the field of wireless communications owing to their great potential to design new-type wireless communication systems and reconfigure the EM environment and wireless channels [36–45]. In the wireless communication area, programmable metasurfaces are also known as reconfigurable intelligent surfaces (RISs) or intelligent reflecting surfaces (IRSs). In [44], transparent metasurfaces were designed to enhance the desired signals and block unwanted signals, but they are passive and cannot dynamically reconfigure the signals. In [39], an RIS was designed to enhance the signal coverage and eliminate signal blind areas by adjusting the direction of the main lobe, addressing the weakness in signal areas. However, the aforementioned metasurfaces and RISs applied in the wireless communication community are only operated in the S and C bands. With the advancement of communication technology, millimeter-wave bands are being explored for mobile wireless communications [46–51], in which the programmable metasurface should exhibit more advantages theoretically compared with conventional technologies. However, as the operating frequency increases, the size of the meta-elements will decrease, making it more difficult to design, manufacture and integrate them with tunable devices, leading to limited research in this area in the literature. In [52], a time-domain digital coding metasurface working in the millimeter-wave band was designed with a 1-bit phase-modulation capability. The metasurface was at the core of the construction of a new schematic 256 QAM millimeter-wave wireless communication transmitter. In [53], a wideband 1-bit RIS was designed to mitigate the effects of signal blind spots in millimeter-wave coverage areas. Compared to 1-bit phase-modulation metasurfaces, multi-bit phase-modulation metasurfaces offer higher gain, improved beamforming precision and broader beam coverage, although they come with increased hardware complexity. In [54], a 2-bit transmissive RIS design with a 90° digital phase shifter and a 1-bit vertical current reversible dipole was presented to work in millimeter-wave bands, which aids wireless communications. However, low integration, high profile and bulky feed structures make most of the above metasurfaces inflexible for applications. Therefore, these implementations suffer from one or more performance defects, such as low channel gain, narrow beam-scanning range, low beamforming resolution and a high side-lobe level. Thus, designing large-scale multi-bit metasurfaces that work in the millimeter-wave bands and realizing precise and wide beam scanning in free space are of great significance and worthy of further investigation.

**Figure 1.**
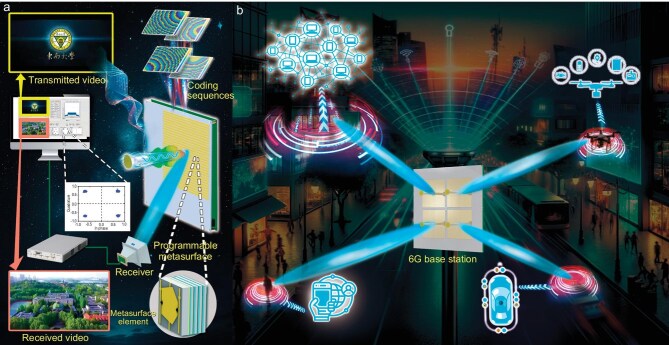
Illustration of the new-type smart beamforming programmable metasurface system. (a) Conceptual diagram of the beamforming system operation process. (b) Multi-user application scenario with four 30 × 30 arrays.

In this paper, we propose a 30 × 30 2-bit millimeter-wave programmable metasurface system for base station application with precise and wide 2D beamforming characteristics. We further construct a multi-user indoor scenario to assist the base station in realizing four-stream information transmissions by using four 30 × 30 arrays. The size of the metasurface array is ∼12.5 *λ*^2^, comprising 30 × 30 meta-elements and 1800 PIN diodes. Each meta-element incorporates two embedded PIN diodes to achieve 2-bit phase-modulation capability. A four-layer control network for tunable devices is implemented to independently adjust the EM phase response of each meta-element. In total, 1800 control ports are deployed in this metasurface to link the corresponding control board. The control board comprises an FPGA core and 38 power distribution chips, equipped with 1800 independent output ports to control all the PIN diodes. One dish-cone antenna is integrated into the center of the metasurface to serve as the feeding source, realizing the integration of transmission and manipulation. A conceptual diagram of the beamforming system operation process is shown in Fig. 1a. The simulation and experimental results indicate that the designed large-scale metasurface possesses 2-bit phase-modulation capability in the millimeter-wave band and achieves a wide beam-scanning range in free space (–70° ≤ *θ* ≤ 70°, 0° ≤ *φ* ≤ 360°) with a gain of 23 dBi. Finally, the proposed metasurfaces help the millimeter-wave base station to realize real-time information transmission of multi-users with different directions in a realistic indoor scenario. The experimental results demonstrate that the new beamforming base station system can intelligently enhance or attenuate signals in specific target areas. We believe the proposed programmable metasurface offers valuable insights for the development and application of smart wireless communication systems, contributing to the advancement of next-generation mobile communication technologies.

## RESULTS

### Coding method for programmable metasurface beamforming

A phased array is advanced antenna technology that electronically controls the phase of individual elements within an antenna array, allowing directional beam steering without the need for physical movement of the antenna. By precisely controlling the phase of the transmitted or received signals, the phased array can rapidly and accurately change the beam direction to point in different directions or track moving targets. Here, the programmable metasurfaces can be regarded as a type of 2D phased array. By appropriately arranging the phase distribution on the surface, specified beamforming can be achieved. Assuming that the proposed meta-element has an *N*-bit phase-modulation ability, various functionalities can be achieved by configuring different coding sequences based on classical phased array theory. For a typical metasurface comprising *N* × *M* periodically arranged meta-elements, we define *A*(*m, n*) and *ф*(*m, n*) (*n* = 1, …, *N* and *m* = 1, …, *M*) to represent the amplitude and phase of the reflection coefficient of the (*m, n*)-th meta-element located at the *m*-th row and *n*-th column. Under the normal incidence of plane EM waves, the scattering pattern *F*(*θ, φ*) of the metasurface can be calculated as:


(1) \begin{eqnarray*}
&& F\!\left( {\theta ,\varphi } \right) = \sum\limits_{m = 1}^M {\sum\limits_{n = 1}^N {E\!\left( {\theta ,\varphi } \right)}}\cdot A\!\left( {m,n} \right) \cdot \exp \\
&&\left( { - i\left( \begin{array}{@{}l@{}} \phi \left( {m,n} \right)\\
+ (n - 1) \cdot k \cdot dx \cdot \sin (\theta ) \cdot \cos (\varphi )\\
+ (m - 1) \cdot k \cdot dy \cdot \sin (\theta ) \cdot \sin (\varphi ) \end{array} \right)} \right),\\
\end{eqnarray*}

where *θ* and *φ* represent the angles of elevation and azimuth, respectively. *E*(*θ, φ*) is the far-field pattern of the meta-elements, *k* is the wave number in free space, and *dx* and *dy* are the side lengths of the meta-element along the *x*-axis and *y*-axis, respectively. Here, we assume that the values of *E*(*θ, φ*) and *A*(*m, n*) are normalized to *E*(*θ, φ*) = cos(*θ*) and *A*(*m, n*) = 1. Based on Equation (1), the scattering field and main-lobe direction of the metasurface can be calculated once the coding sequence is fixed.

**Figure 2.**
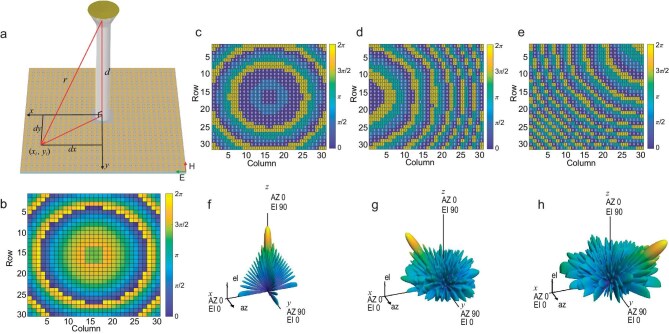
Schematic diagram of the 2-bit beamforming metasurfaces. (a) Schematic diagram of calculating the distance in the 2-bit metasurfaces. (b) Initial phase distribution of the designed metasurface. (c–e) Coding sequence realized by the encoding method corresponding to the main lobe pointing at φ = 0°, θ = 0°, φ = 0°, θ = –30° and φ = 45°, θ = 45°, respectively. (f–h) Calculated 3D scattering patterns at 25 GHz corresponding to the above coding sequences, respectively.

Furthermore, the phase distribution of the meta-elements in the metasurface array corresponding to the main-lobe direction angle can be expressed as:


(2) \begin{eqnarray*}
\Phi (m,n) &=& - k \cdot ( {{x_i} \cdot \sin {\theta _0} \cdot \cos {\varphi _0} + {y_i} \cdot \sin {\theta _0}}\\
&&\cdot \sin {{\varphi _0}} ) + {\phi _0}\big( {m,n} \big),
\end{eqnarray*}

where *Ф*(*m, n*) is the phase of the (*m, n*)-th meta-element, (*x_i_, y_i_*) represents the position of the (*m, n*)-th meta-element in the Cartesian coordinate system, (θ_0_, φ_0_) is the main-lobe direction angle, and ф_0_(*m, n*) corresponds to the initial phase of the (*m, n*)-th meta-element, influenced by the distance between the meta-elements and the phase center of the feeding source. As shown in Fig. 2a, *x* and *y* represent the distance from the (*m, n*)-th meta-element center to the origin point along the *x* and *y* directions, respectively. *d* represents the distance between the origin point and the phase center of the feeding source; *r* represents the path length from the feeding source to the (*m, n*)-th meta-element center, where $r = \sqrt {{x^2} + {y^2} + {d^2}} $. The initial phase of the (*m, n*)-th meta-element is expressed by:


(3) \begin{eqnarray*}
{\phi _0}( {m,n} ) = \frac{{2\pi r}}{\lambda },
\end{eqnarray*}

where *λ* is the working wavelength in free space. The phase distribution of the metasurface is set to be continuous. To achieve beam scanning with the designed metasurface, the phase needs to be discretized into *N*-bit by using the formula:


(4) \begin{eqnarray*}
\textit{Code}\left( {m,n} \right) = \left\{ {\begin{array}{@{}*{1}{c}@{}} {0,}\\ {1,}\\ { \vdots ,}\\ {{2^N} - 1,} \end{array}
\begin{array}{@{}*{1}{c}@{}} {0 \le \Phi \left( {m,n} \right) < {{2 \cdot \pi } / {{2^N}}}}\\
{{{2 \cdot \pi } / {{2^N}}} \le \Phi \left( {m,n} \right) < 2 \cdot {{2 \cdot \pi } / {{2^N}}}}\\ \vdots \\ {\left( {{2^N} - 1} \right) \cdot {{2 \cdot \pi } / {{2^N}}} \le \Phi \left( {m,n} \right) < 2 \cdot \pi } \end{array}} \right.,
\end{eqnarray*}

where *Code*(*m, n*) represents the phase code of the (*m, n*)-th meta-element at the main-lobe pointing angle of (θ_0_, φ_0_).

Assuming a 2-bit phase-modulation programmable metasurface arranged periodically by 30 × 30 meta-elements, the periodic length is 5 mm and the distance from the origin point to the phase center of the feeding source is 110 mm. At 25 GHz, we can derive the coding sequences for various beam configurations and their corresponding far-field patterns based on Equations (1)–(4). The initial phase profile from the feeding antenna to the metasurface is calculated and shown in Fig. 2b. Figure 2c–e respectively illustrates the coding sequence distributions to form the main lobes pointing at (*φ* = 0°, *θ* = 0°), (*φ* = 0°, *θ* = –30°) and (*φ* = 45°, *θ* = 45°) and the corresponding scattering patterns are shown in Fig. 2f–h.

### Large-scale 2-bit millimeter-wave programmable metasurface prototype

**Figure 3.**
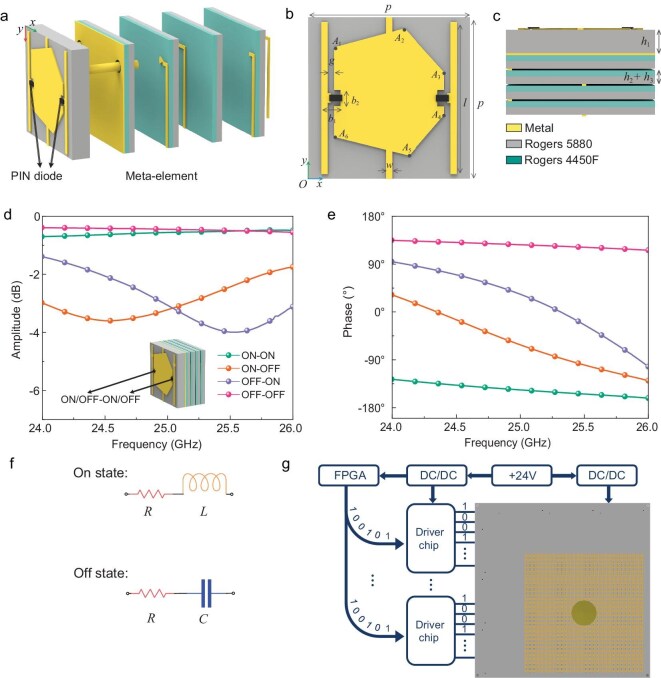
Introduction of the meta-element and control board. (a) Explored structure of the designed 2-bit phase-modulation meta-element. (b) Front view of the meta-element. (c) Side view and materials of the meta-element. (d) Simulated EM amplitude responses of the designed meta-element at four different switching states in the CST Microwave Studio. (e) Phase responses. (f) Equivalent circuit of the PIN diodes in ON state or OFF state. (g) Operating mechanism of the proposed control board.

We design a 2-bit meta-element that works in the millimeter-wave band by using a novel design method based on the multiple-ports model [55]. The design target is set to obtain 2-bit reflective phase responses (four phase states with 90° intervals) and lower reflective losses (losses of <3 dB) in 24–26 GHz. The concepts of phase-modulation metasurfaces are detailed in Supplementary Materials Section S1. The final structure of the meta-element is shown in Fig. 3a. It consists of one metal patch layer, one metal sheet layer, four control line layers and five dielectric layers. The metasurface array includes 30 × 30 meta-elements with detailed geometrical parameters, as shown in Fig. 3a–c (for details, see Methods). The PIN diodes used in the meta-elements are MADP-000907-14020w, manufactured by MACOM Inc. The impedance model of the diode is extracted from its data sheet with a numerical fitting method, as shown in Fig. 3f, where two equivalent circuits representing the two states of the PIN diode are presented. In the ON state, the diode is modeled as a resistor and an inductor in series with values of *R* = 4.6 $\Omega $ and *L* = 0.204 nH. In the OFF state, the diode is modeled as a resistor (*R* = 6.5 $\Omega $) and a capacitor (*C* = 0.063 pF) in series.

The phase-modulation performance of the meta-element is simulated in the commercial EM simulation software, CST Microwave Studio. In the simulation set-up, the frequency domain solver is used to obtain the reflection coefficients of the meta-element for its four switching states of the two diodes. The simulation frequency is set at from 24 to 26 GHz, with the boundary condition set to ‘unit cell’ to simulate the meta-element with an infinite periodic situation. The orientation of the electric field is aligned with the direction of the PIN diodes to ensure their proper functionality. Following the simulation, the results of the meta-element are shown in Fig. 3d and e. The reflection phases cover a 90° interval in the range of 24.5–25.5 GHz under four switching states and the reflection losses of all the cases are <4 dB within this frequency band. Here, we define ‘0’, ‘1’, ‘2’ and ‘3’ to denote the ‘ON–ON’, ‘ON–OFF’, ‘OFF–ON’ and ‘OFF–OFF’ states, and they correspond to the four reflective phases of 0°, 90°, 180° and 270°, respectively. The corresponding experimental results are shown in Section S2. According to the aforementioned results, the designed meta-element exhibits 2-bit phase-modulation ability in the millimeter-wave band, along with low loss properties.

**Figure 4.**
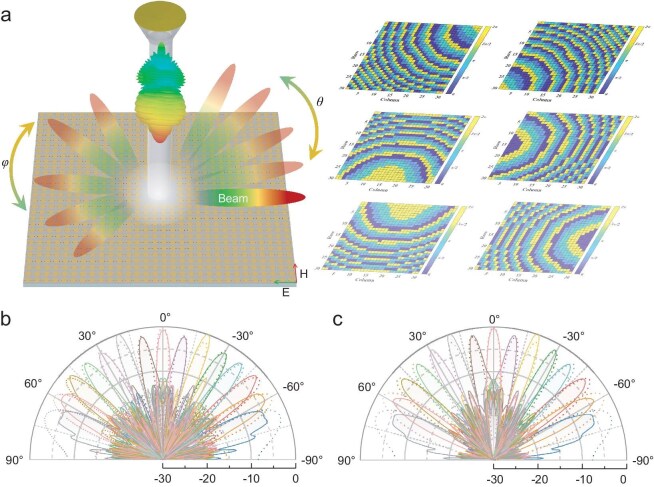
Calculated and simulated beamforming results of the meta-array. (a) Schematic of spatial beam scanning achieved by the designed beamforming system. (b, c) Calculation (dash lines) and simulation (solid lines) results of the metasurface beam scanning from –70° to 70° with 10° intervals of the elevation angle in the plane φ = 0° (b) and φ = 45° (c) at 25 GHz.

Here, we use a type of dish-cone antenna as the feeding source, which can realize back radiate with a wide operating band, good omnidirectionality and high gain. The detailed information on this designed antenna is described in Section S3. As the angle of the incident wave from the dish-cone antenna to the meta-elements is not consistent, we simulate the meta-element in CST for transverse electric and transverse magnetic oblique incident waves ranging from 0° to 40° with an interval of 10°. The simulation results demonstrate the angle-insensitive capability of the designed meta-element. Detailed analysis is provided in Section S4. Owing to the equipment requirement of the feeding source, 4 × 4 meta-elements located in the center of the metasurface are removed to provide the space. Figure 3g demonstrates a schematic diagram of the large-scale programmable metasurface with the integration of the meta-array, feeding network and control board. The space on the left and bottom sides of the metasurface array is reserved for the connection of control signal output ports. The control signals are generated by using a customized control board, which is composed of an FPGA microchip, 38 power driver chips and 1800 independent voltage output ports. A +24-V DC voltage is transformed into the operating voltages of various devices, such as FPGA, drive chips and tunable devices, by utilizing multiple DC/DC switch power-supply circuits, thereby maximizing the power-supply efficiency. In addition, the drive-state switching is consistent based on the ability of the FPGA for parallel computing and the synchronous trigger instruction of the driver chips. By calculating the corresponding EM phase response states of the meta-elements, the output voltages are adjusted to control the working states of the PIN diodes, achieving beamforming in the system. The aforementioned three parts constitute the complete hardware architecture of the beamforming system. Combined with the beamforming coding theory, this system enables the spatial scanning capability in the millimeter-wave band, as shown in Fig. 4a.

To validate the beamforming performance of the system, we compute the coding sequences and corresponding far-field patterns, respectively, where the main lobe points at the *φ* = 0° and *φ* = 45° planes, *θ* from –70° to 70° with a 10° interval, as shown in Fig. 4b and c with dash lines. It is noteworthy that the calculation process fails to take into account the coupling among the meta-elements, and assumes the reflection amplitudes and radiation patterns of the elements to be in an ideal state. Furthermore, we conduct additional simulations of the far-field patterns for these calculated coding sequences by using the commercial EM simulation software, CST Microwave Studio. The simulated results are represented by solid lines in Fig. 4b and c. There are discrepancies between the simulation and calculation results. The main reason is that the calculation provides idealized solution outcomes and ignores the influence of the central meta-elements. Nevertheless, the direction of the main lobe still aligns with the simulation and calculation results, validating the beam-scanning performance of our proposed system.

### Experimental verification

**Figure 5.**
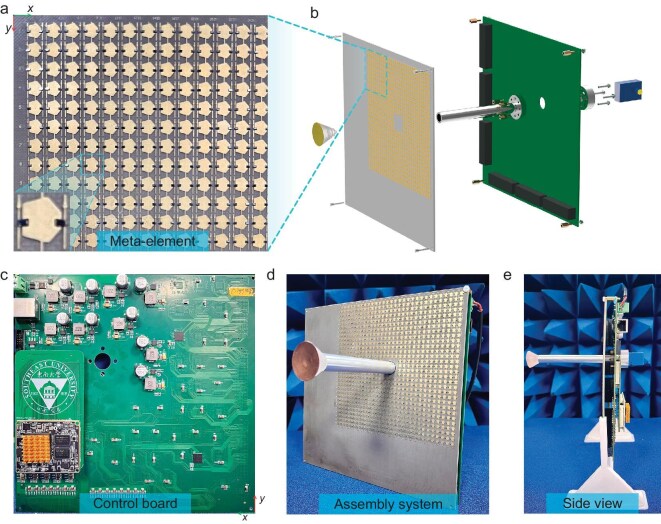
System architecture and prototype of the system. (a) Picture of the fabricated metasurface made of 30 × 30 2-bit phase-modulation meta-elements. (b) Schematic diagram of the disassembly model of the system. (c) Photograph of the fabricated control board with 1800 output ports. (d) Assembly picture of the proposed beamforming system. (e) Side view of the system.

To experimentally validate the performance of the proposed beamforming system, we manufacture all system components by using the standard printed circuit board technology and mold manufacturing techniques. As shown in Fig. 5a, the overall size of the entire metasurface board is 217 × 217 mm^2^ (18 × 18 ${\lambda}^2$) and the actual size allocated for arranging the 30 × 30 meta-elements is 150 × 150 mm^2^ (12.5 × 12.5 ${\lambda}^2$). The remaining area is allocated for control networks and connection ports. Figure 5b demonstrates the disassembly diagram of the metasurface array, feeding antenna and control board. The size of the designed control board matches the metasurface to ensure the integral assembly, as shown in Fig. 5c. The three parts are finally assembled with some studs, constructing the beamforming system as shown in Fig. 5d and e.

#### Beam-scanning validation

**Figure 6.**
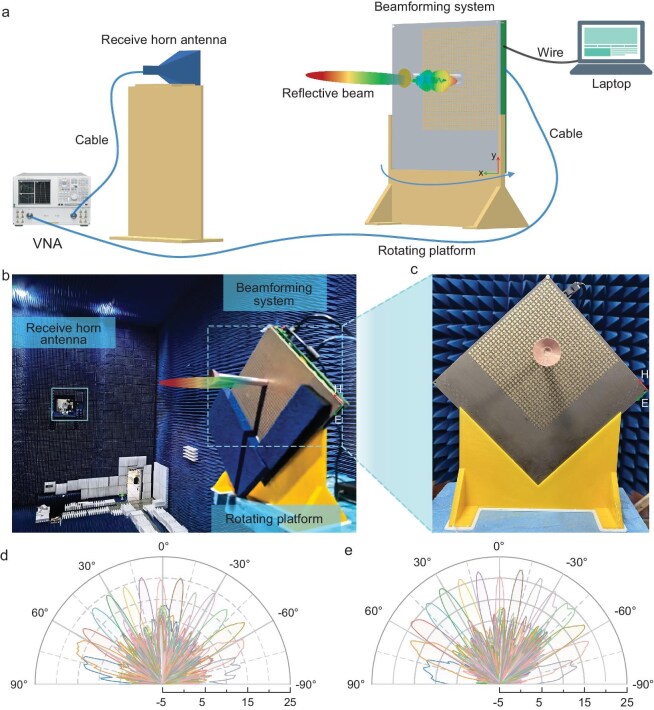
Beamforming experimental set-up and results of the system. (a) Schematic view of the experimental set-up to measure the far-field scattering patterns of the proposed system. (b) Configuration of the measurement set-up. (c) Photograph of the experimental beamforming system. (d, e) Experimental results of the metasurface beam scanning from –70° to 70° with 10° intervals of elevation angle in the plane *φ* = 0° (d) and *φ* = 45° (e) at 25 GHz.

The beam-scanning validation experiment is conducted in the microwave anechoic chamber. The measurement schematic of the experiment is shown in Fig. 6a, where the beamforming system is securely mounted on a rotating platform. A vector network analyser is connected to both the rectangular waveguide port of the system and a standard gain horn antenna. The waveguide supplies the excitation signals, while the horn antenna is used to receive the signals. To ensure far-field conditions at the receiver, the distance between the horn antenna and the system should be >${{2{D^2}} / \lambda }$, where *D* is the largest effective size of the metasurfaces and *λ* is the operating wavelength. In this set-up, the horn antenna is positioned 10 m away from the metasurface. The centers of the horn antenna and the metasurface lie in the same plane and are positioned parallel to each other. The orientation of the electric field of the EM waves, excited by the feeding source, is aligned with the orientation of the PIN diodes. The measurement set-up is shown in Fig. 6b and c.

The measured scattering patterns of the beamforming system are shown in Fig. 6d and e. The experimental results demonstrate that the reflected beam can scan from *θ* = –70° to *θ* = 70° at *φ* = 0° and *φ* = 45° planes. The gain of the beam reaches ∼23 dBi after normalizing with a standard horn antenna and the side-lobe level is about –12 dB. The deviation between the simulated and experimental results arises from manufacturing tolerances and direct radiation from the feeding source. Nevertheless, the results are still in good agreement in terms of wide beam-scanning angles. The good beam-scanning performance demonstrates the effectiveness of the proposed beamforming system. The potential challenges and limitations in scaling up such metasurface systems for real-world 6G base station deployments are discussed in Section S6.

#### Smart base station wireless communication demonstrations

##### Single-stream wireless communication.

**Figure 7.**
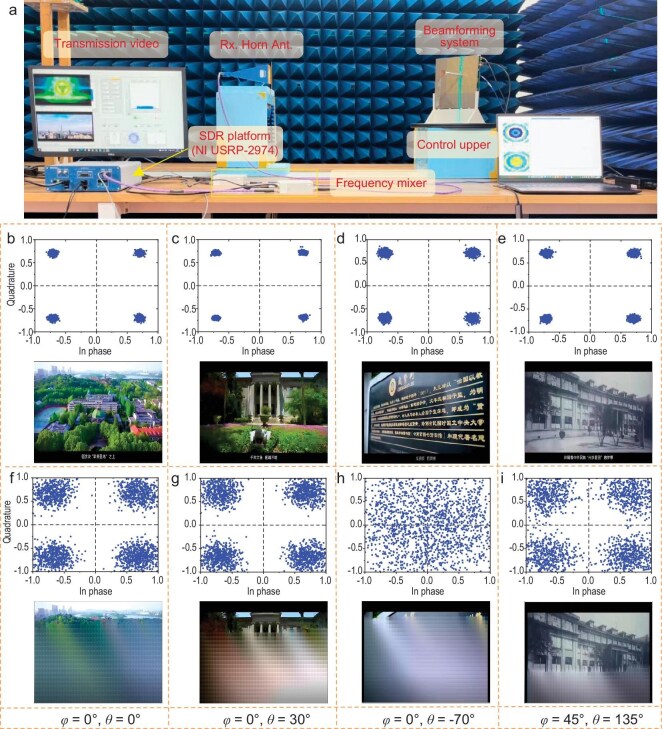
Experimental set-up and results of wireless communications with the system. (a) Scenario of the experimental set-up to demonstrate the proposed system-aided wireless communications. (b–e) Constellations and transmitted video screenshots of the receivers located at (*φ* = 0°, *θ* = 0°), (*φ* = 0°, *θ* = 30°), (*φ* = 0°, *θ* = –70°) and (*φ* = 45°, *θ* = 135°) with the aid of the beamforming system, respectively. (f–i) Constellations and transmitted video screenshots of the receivers located at the same position without the aid of the beamforming system.

For illustrating the potential of the proposed prototype in the application of a smart 6G base station, we take the proposed system to assist a millimeter-wave base station and validate its performance of wireless communication in a realistic indoor scenario. The wireless communication experiments are conducted with a software-defined radio (SDR) platform (NI USRP-2974). The measurement schematic of the demonstration is shown in Fig. 7a, where the beamforming system is securely mounted on a rotating disk. Here, we use the proposed system to replace the traditional high-cost and complex radio-frequency front end to connect the SDR platform. A standard gain horn antenna operating at 25–29 GHz is connected to the SDR platform as the user antenna. Two frequency mixers are linked between the SDR platform, the proposed system and the horn antenna to expand the working band to the target millimeter-wave band. A video is converted into bitstreams and transmitted by the beamforming system through quadrature phase shift keying (QPSK) modulation. The user is positioned 1 m away, sequentially at four different positions ((*φ* = 0°, *θ* = 0°), (*φ* = 0°, *θ* = 30°), (*φ* = 0°, *θ* = –70°) and (*φ* = 45°, *θ* = 135°)) for demonstration purposes. The SDR platform then demodulates the received signals to recover the transmitted video (for details, please refer to Video S1). Figure 7b and f shows the constellation diagrams of the beamforming system when the system and the receiver center are aligned at *φ* = 0°, *θ* = 0°. However, the video can only be transmitted smoothly when the beam of the system is directed at *φ* = 0°, *θ* = 0°. This result is consistent with the far-field radiation pattern experiment, which shows that, when the beam is directed at 0°, the energy in 0° directions is relatively high. Furthermore, we test the wireless communication performance when the user is at positions *φ* = 0°, *θ* = 30°, *φ* = 0°, *θ* = –70° and *φ* = 45°, *θ* = 135°, both with and without the system operating at corresponding pointing angles. The experimental results are shown in Fig. 7c–e and Fig. 7g–i, respectively. Clear constellation diagrams and smooth, uninterrupted video transmission can be obtained only when the system forms its main beam to the user. The signal energy boosted in the specified direction guarantees communication speed and data integrity. This verifies that the proposed system has an excellent beamforming capability to act as good base station auxiliary equipment that can cover a wide angle range of ±70° in the upper half-space.

##### Four-stream wireless communication.

**Figure 8.**
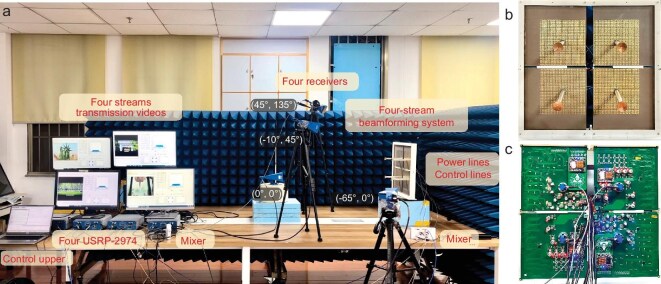
Prototype and experimental set-up of the four-stream beamforming system. (a) Scenario of the experimental set-up to demonstrate the four-stream beamforming system-aided wireless communication. (b) Front view and (c) back view of the four-stream beamforming system.

Furthermore, we construct a four-stream beamforming system based on two 30 × 30 arrays and two mirror 30 × 30 arrays, as shown in Fig. 8b. The back view of the system is shown in Fig. 8c. To demonstrate its performance in multi-user communications, we take the four-stream system as the core and investigate its communication-assisted ability in a realistic indoor scenario. The measured environment is similar to the aforementioned single-stream wireless communication-assisted experiment, as shown in Fig. 8a. The difference is that four SDR platforms (NI USRP-2974) and four receiving antennas are employed here to accomplish the transmission of four independent video streams. The transmitted method is still QPSK modulation. Four users are placed at angles (*φ* = 45°, *θ* = 135°), (*φ* = 0°, *θ* = 0°), (*φ* = –10°, *θ* = 45°) and (*φ* = –65°, *θ* = 0°) relative to the center of the four-stream beamforming system. The video can only be transmitted properly when the beam pointing angle of the stream is facing these directions, and vice versa. In addition, when the system operates under the specified beamforming conditions, it can enable simultaneous and independent transmission of four video streams (for details, please see Video S2). This experiment demonstrates the performance of the multi-user communication-assisted set-up, highlighting the potential to enhance the channel capacity of 6G base stations assisted by programmable metasurfaces. The independent and reconfigurable capabilities in manipulating the propagation directions of four streams have facilitated the smart communication of the new types of base stations. We summarize the properties of the proposed beamforming system in Table 1. Furthermore, the proposed system also offers a practically deployable hardware platform for future intelligent communications.

**Table 1.** Parameters of the proposed beamforming system.

Metasurface properties		Control-board properties		System properties	
Unit number	884	Control method	Serial-to-parallel conversion	Beamforming (*θ*)	–70° to 70°
PIN number	1768	Switching rate	<625 KHz	Beamforming (*φ*)	0°–360°
Port number	1800	FPGA number	1	Gain (one piece)	23 dBi
Unit size	0.43 *λ*	Power chips	38	Side-lobe level	>10 dB
Working bands	24.5–25.5 GHz	Block RAM	1.8 Mbits	Multiple users	Four streams
Phase precision	2-bit	Control ports	1824	Power consumption	12 W

## CONCLUSION

We propose a comprehensive, large-scale 2-bit millimeter-wave programmable metasurface system for smart base station applications with precise and wide 2D beamforming characteristics. The system comprises a feeding source, a programmable metasurface and a control board. The feeding source is composed of a dish-cone antenna operating in the millimeter-wave band. The programmable metasurface is constructed with 30 × 30 meta-elements arranged periodically and the overall structure includes 1800 control lines. The designed meta-element features 2-bit phase modulation, achieved by independently controlling the PIN diodes. The control board includes the FPGA core, power-drive chips and 1800 output ports, capable of supplying precise tunable voltages to the PIN diodes. Using the programmable metasurface beamforming encoding method, we derive the coding sequences for various main-lobe angles and their corresponding far-field scattering patterns. Additionally, we calculate and simulate the beam-scanning performance of the metasurface with the azimuth angle at *φ* = 0°, *φ* = 45°, and elevation angle *θ* from *–7*0° to 70°. The experiment results show high consistency with the calculations and simulations, successfully validating the good performance of the proposed system. Additionally, we respectively construct a single-stream system and a four-stream system for 6G smart base station applications in a realistic indoor scenario. The proposed beamforming system integrates the feeding source, programmable metasurface and control board, efficiently realizing the functions of both the transmitter and the beamformer. Furthermore, the system can enhance or attenuate signals in specific directions, leveraging its beamforming capabilities. The good performance indicates its significant applications as a base station auxiliary equipment working in the millimeter-wave band and suggests its potential to inspire the development of new wireless communication technologies.

## METHODS

### Meta-element design details

As shown in Fig. 3a–c, the upper layer of the meta-element is the metal pattern layer, in which a quasi-hexagonal structure is deployed in the center, and two PIN diodes are embedded in the metal gaps. Three strip control lines are connected to the two diodes to supply the required bias voltages. The middle control line serves as the common terminal, while the others function as the control terminals of the diodes. The substrate material is Rogers 5880, which has a low loss in the high-frequency band. Rogers 4450F is employed as the glue layers to bond the substrates and metal layers. A metal sheet is placed under the first substrate to reflect incident EM waves. The thickness of all of the copper metal layers is 0.018 mm. Two metal vias penetrate all layers, combining the control lines to provide bias voltages for the PIN diodes. At least four layers are required here for control network deployment due to the large number of diodes and limitations of the meta-element size and manufacturing process. Two sector-shaped patches are placed on the first control line layer, connecting two vias to construct a low-pass filter, achieving isolation between alternating and direct currents. The final detailed structure geometric dimensions are listed as follows: *p* = 5 mm, *l* = 4.7 mm, *g* = 0.2 mm, *w* = 0.2 mm, *b*_1_ = 0.6 mm, *b*_2_ = 0.5 mm, *h*_1_ = 0.787 mm, *h*_2_ = 0.2 mm, *h*_3_ = 0.25 mm. We use coordinates to describe the geometric dimensions of the quasi-hexagonal structure in the Cartesian coordinate system, as shown in Fig. 3b. We define *O* as (0, 0), then *A*_1_, *A*_2_, *A*_3_, *A*_4_, *A*_5_ and *A*_6_ are (0.8, 4.1), (3, 4.7), (4.2, 3.3), (4.2, 2), (3.1, 0.7) and (0.8, 1.3), respectively. The side view and the types of materials in different layers are shown in Fig. 3c.

## Supplementary Material

nwaf017_Supplemental_Files

## Data Availability

The data that support the plots within this paper and other findings of this study are available from the corresponding author upon reasonable request.
