# Supplementary material for: A smart millimeter-wave base station for 6G application based on programmable metasurface
Source: Natl Sci Rev. 2025 Jan 16;12(4):nwaf017. doi: 10.1093/nsr/nwaf017 (PMC11892553; doi:10.1093/nsr/nwaf017)
Supplement: nwaf017_Supplemental_Files [file nwaf017_supplemental_files.zip › Proof_Supplementary_materials.pdf]

Supplementary Materials for

A Smart Millimeter-Wave Base Station for 6G Application Based on Programmable Metasurface

Jun Wei Zhang *et al*.

Corresponding author: Jun Yan Dai, junyand@seu.edu.cn, Qiang Cheng, qiangcheng@seu.edu.cn, and Tie Jun Cui, tjcui@seu.edu.cn

This PDF file includes:

Section 1 to Section 6

1: Concepts of Phase Modulation Metasurfaces

2: Experiment Results of the Designed Metasurface Performance

3: Introduction to the Feeding Source

4: Verification of Meta-Elements' Angle-Insensitive Abilities

5: Introduction to the Control Board

6: The Potential Challenges and Limitations in Scaling up Such Metasurface Systems

Figs S1 to S3

Section 1: Concepts of Phase Modulation Metasurfaces

For a phase modulation metasurface, the phase responses of the meta-elements can be controlled, while the amplitude remains unaltered. A 1-bit phase modulation metasurface can use "0" and "l" to define two phase states, such as 0° and 180°. The spatial arrangement of "0" and "l" meta-elements in the metasurface allows control of reflected EM waves, thereby reconfiguring the EM environments. The concept of phase modulation metasurfaces can be extended from 1-bit to N-bit ($N > 1$). The N-bit phase modulation metasurfaces consist of 2^N coding states with a phase interval of $360/2^N$. The phase response of ith-state Φ_i is defined by

$$\Phi_i = \frac{2\pi \cdot i}{2^N} + \varphi_0, \tag{1}$$

where φ_0 is the initial phase and $i = 0, ..., 2^N\text{-}1$.

For example, one phase modulation operating mechanism of the designed meta-elements is to achieve an abrupt phase change by adjusting the resonant operating frequency of the metasurface. By integrating the varactor diodes, the continuous adjustment of capacitance can be realized, and then the resonant frequency can be continuously varied according to this formula

$$f = \frac{1}{2\pi\sqrt{L \cdot C}}, \tag{2}$$

where L and C represent the equivalent inductor and capacitor of the meta-elements. By tuning the capacitance values of the varactor, the desired phase gradient change can be achieved. Then the design of the metasurface with a certain phase interval can be realized. Finally, by applying the appropriate bias voltages to the fabricated metasurfaces in practice, the desired EM phase responses can be measured.

Section 2: Experiment Results of the Designed Metasurface Performance

We measure the reflected coefficients of the design metasurface array in the anechoic chamber. The experimental setup is shown in Fig. S1a. One lens antenna operated at 18-40 GHz is linked to the vector network analyzer (VNA). The metasurface array is placed directly below the lens antenna. The time gate approach is employed to reduce the influence of multipath reflection. The experimental results are shown in Fig. S1b and c. The reflection phases cover a 90° interval in the 25-26 GHz under four switching states, and the reflection losses of all the cases are about 6 dB at the frequency of 25.5 GHz. The primary reasons for the measured higher loss and frequency shift of the system compared to the simulation results can be attributed to the processing tolerances, deviations of material and device parameters, as well as the truncation effect of the finite meta-array.

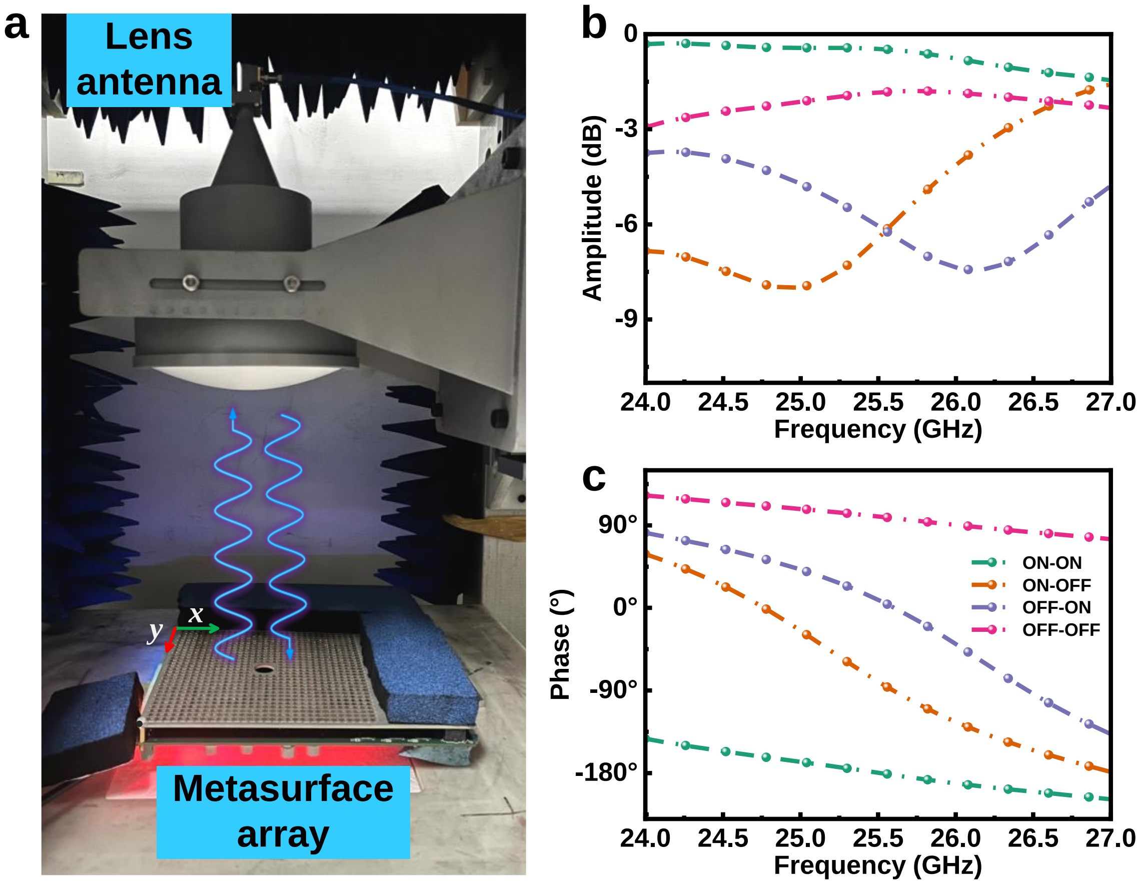

Fig. S1 The experimental EM responses of the designed meta-array. (a) Experimental setup of the meta-array. (b) Measured EM amplitude responses of the designed array at four different switching states. (e) Phase responses.

Section 3: Introduction to the Feeding Source

The structure of the feeding source in the system is shown in Fig. S2a and b. The feeding source is a dish cone antenna, consisting of four parts: the disc cone head, the waveguide coaxial tube, the waveguide-to-coaxial converter, and the millimeter wave band rectangular waveguide. The disc cone head is made of REXOLITE1422 material with the upper part of the head surface covered in copper metal. The waveguide coaxial tube and the waveguide-to-coaxial converter are carefully designed to ensure the propagation of incentive EM waves. The chosen feeder is a BJ260 standard rectangular waveguide, which works in the frequency range from 22 GHz to 33 GHz. To ensure the dish cone antenna operates within the designed operational frequency band, its structural dimensions are optimized and scanned during the design process. The specific geometric dimensions are as follows: d=112 mm, d1=27.52 mm, d2=13.46 mm, d3=9.46 mm. In order to adapt to the meta-array structure and achieve integrated and disassembled assembly design, various tooling design structures have been added to the entire antenna to ensure feasibility and stability in actual usage.

The performance of the designed dish cone antenna is verified through simulation using the

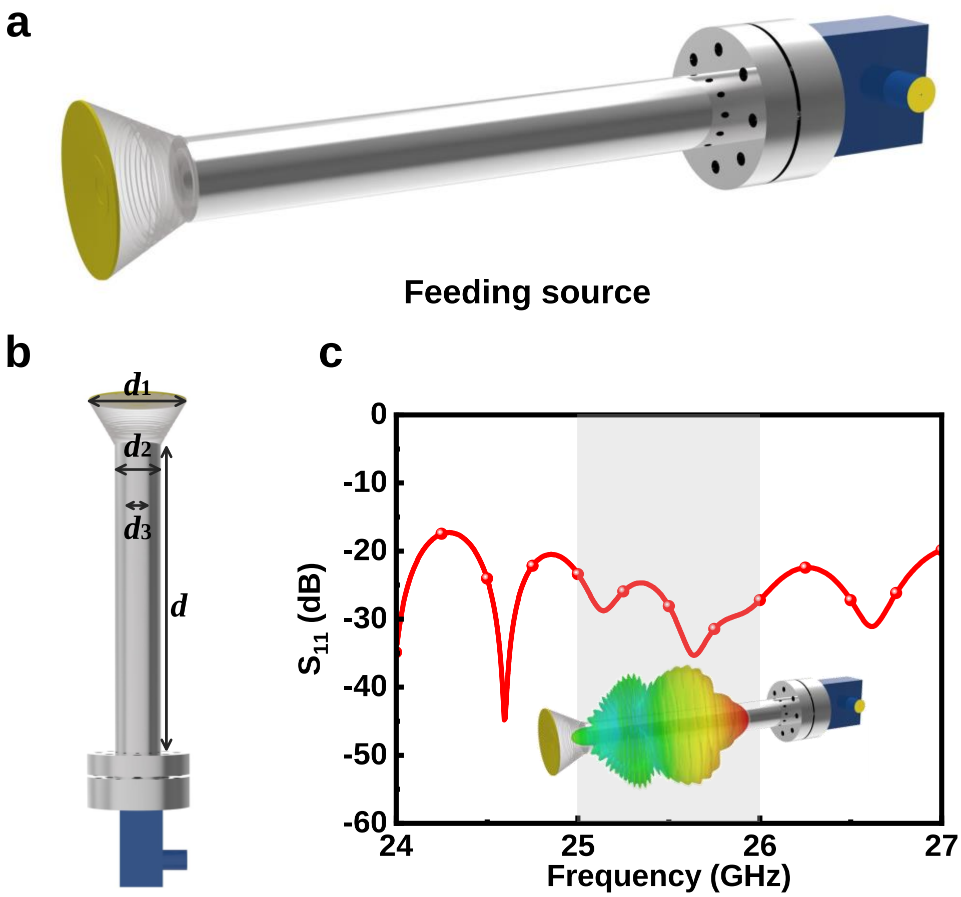

Fig. S2 The introduction of the designed feeding source. (a) Illustration of the structure of the feeding source. (b) The side view of the feeding source. (c) The reflective coefficient S_{11} of the designed dish cone antenna.

commercial simulation software CST Microwave Studio. In the simulation setup, the rectangular waveguide is replaced by a matched waveguide port. The operating frequency is set from 24 GHz to 27 GHz. The simulation result is shown in Fig. S2c, where the reflective coefficient S_{11} is lower than -20 dB in the 25-26 GHz band, as indicated in the gray area. The far-field pattern of the antenna is also shown in Fig. S2c. These results demonstrate that the designed feeding source has the ability to back-radiate while having a wide operating band, good omnidirectionality, and high gain.

Section 4: Verification of Meta-Elements' Angle-Insensitive Abilities

Owing to the distance between the disc cone antenna and the metasurface not satisfying the far-field condition, the EM wave arriving at the metasurface cannot be considered as a plane wave. As a result, the arrival phase and incident angle of the EM wave on each meta-element are different. To verify the EM response performance of the meta-elements under various incident waves, we simulate the corresponding meta-elements using commercial simulation software CST. The distance between the feeding source and metasurface is denoted as *d*, as shown in Fig. S3a, and the distance from the farthest meta-element center to the phase center of the feeder is *r*. The angle of the incident wave from the feeder to this farthest meta-element is $\alpha = \arctan(\sqrt{dx^2 + dy^2} / d)$. We calculate the value of α is about 39°, as shown in Fig. S3b. Therefore, we perform EM simulations of the meta-element in CST for TE and TM oblique incident waves ranging from 0° to 40° with an interval of 10°. The polarization direction is always parallel with the direction of the diodes. The simulation results in Fig. S3c-f show that the EM response of the meta-element remains essentially unchanged at large oblique angles, while maintaining stable 2-bit phase responses.

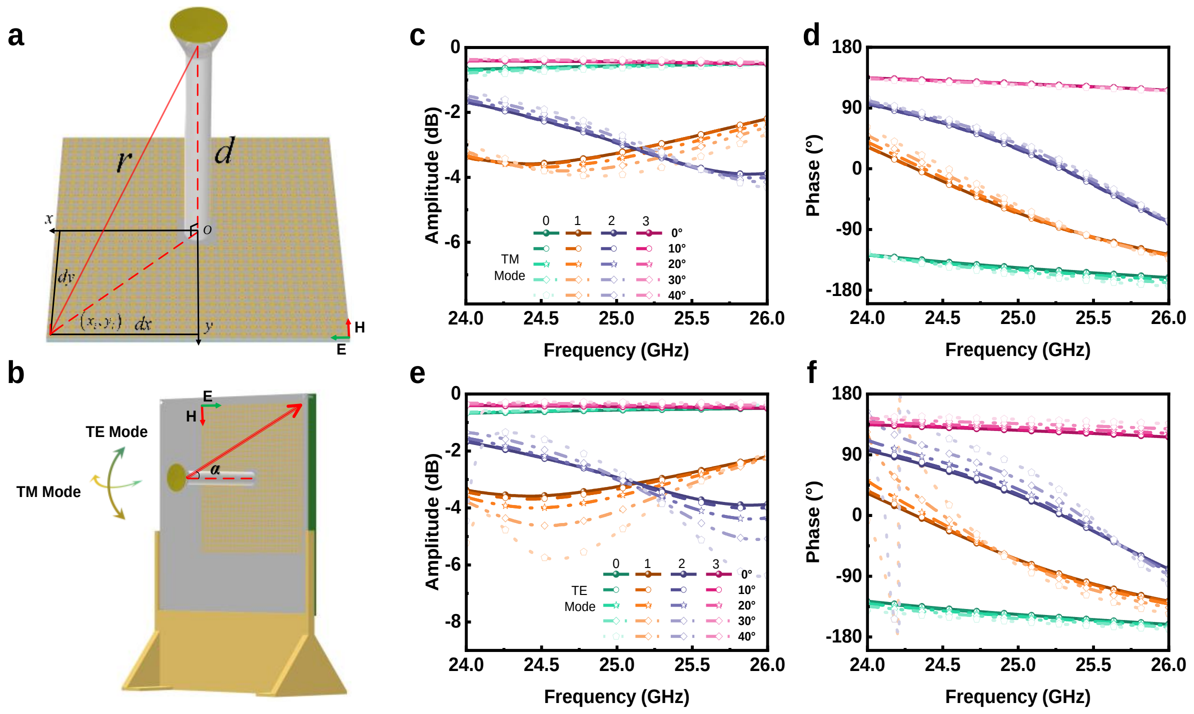

Fig. S3 The simulated results of the meta-element under oblique incident waves. (a) Schematic diagram for calculating the incident angle of the farthest meta-element. (b) The schematic diagram of the oblique incident wave. (c) Reflective amplitude of the meta-element under the TM mode oblique incident wave from 0° to 40° with a 10° interval. (d) Reflective phase under TM mode. (e) Reflective amplitude of the meta-element under the TE mode oblique incident wave from 0° to 40° with a 10° interval. (f) Reflective phase under TE mode.

Section 5: Introduction to the Control Board

In order to achieve independent control of the designed meta-elements within such a large meta-array, we ingeniously design a voltage control board featuring 1,800 independent output ports with an FPGA microchip and 38 power driver chips. This setup facilitates precise control of the meta-elements and good integration of the system. A +24V DC voltage is transformed to the operating voltages of various devices, such as a FPGA and drive chips, utilizing multiple DC/DC switch power supply circuits, maximizing power supply efficiency. Moreover, the control board utilizes a serial-to-parallel driving mode, effectively minimizing the number of pins required by the FPGA. The pre-calculated coding information is loaded into the system via a laptop computer, enabling voltage control output ports and thereby controlling the EM phase responses of meta-elements. In addition, the drive state switching is synchronized. The driver code is configured to trigger all driver chips simultaneously, based on the FPGA's ability for parallel computing. All driver chips receive the state-switch trigger instruction from the FPGA at the same time, ensuring that the registers in the driver chips are read synchronously to maintain consistency in the tuning devices' states.

Section 6: The Potential Challenges and Limitations in Scaling up Such Metasurface Systems

1. The increased complexity of hardware. In order to achieve high-precision beamforming in free space, each unit in the array requires an independent control port. As the size of the array increases, the number of control ports will grow dramatically. However, this can be alleviated by employing advanced IC modules which integrate more output pins for element control.

2. The high computational demands. As the meta-array size increases, the computational complexity will also be increased, leading to the growing time cost of the codebook generation. To solve that problem, there are more new schemes for efficient beamforming with the metasurface structures reported in the literature, and AI technologies can also be applied in the training of the coding sequences.

3. The increased power consumption. The power consumption of the base station becomes a serious problem in practical applications. For the new metasurface-based base station system, the power consumption mainly comes from the signal source, the tunable devices, and the controller. It can be found that as the array size increases, the power consumption of tunable devices and controllers also increase as well, but without the T/R modules, the overall power consumption is still far lower than traditional base stations. When new type of diodes with very small currents are introduced in the element design, the total power consumptions will also be dramatically decreased at large scale of the aperture.
